# The role of family planning counselling during maternal and child health services in postpartum modern contraceptive uptake in Ethiopia: A national longitudinal study

**DOI:** 10.1371/journal.pgph.0000563

**Published:** 2022-08-03

**Authors:** Kalayu Brhane Mruts, Gizachew A. Tessema, Amanuel Tesfay Gebremedhin, Jane A. Scott, Gavin Pereira

**Affiliations:** 1 School of Population Health, Curtin University, Perth, Australia; 2 School of Public Health, University of Adelaide, Adelaide, Australia; 3 Wesfarmers Centre for Vaccine and Infectious Disease, Telethon Kids Institute, University of Western Australia, Perth, Australia; 4 Centre for Fertility and Health (CeFH), Norwegian Institute of Public Health, Oslo, Norway; 5 enAble Institute, Curtin University, Perth, Australia; PLOS: Public Library of Science, UNITED STATES

## Abstract

Family planning counselling can help improve the postpartum modern contraceptive uptake. However, studies in Ethiopia indicate inconsistent effects of integrated family planning counselling on postpartum modern contraceptive uptake. This study aimed to determine the extent of family planning counselling and its role in improving postpartum contraceptive uptake among women in Ethiopia. We used the Performance Monitoring for Action (PMA) Ethiopia panel survey data, a community-based prospective cohort study. Randomly selected pregnant women were recruited at the baseline interview and followed by six weeks and six months postpartum. A weighted generalised linear model fitted with a Poisson distribution and a log link function was used to estimate the adjusted relative risk (aRR) and 95% Confidence Interval (CI) of modern contraceptive uptake. The coverages of family planning counselling provision during ANC, prior to discharge and child immunisation were 20%, 27% and 23%, respectively. The modern contraceptive uptakes by six weeks and six months postpartum were 18% and 36%, respectively. Family planning counselling prior to discharge from the facility was associated with increased modern contraceptive uptake by six weeks (aRR 1.25; 95% CI 0.94, 1.65) and six months postpartum periods (aRR 1.07; 95% CI 0.90, 1.27). Moreover, women who received family planning counselling during child immunisation were 35% more likely to use modern contraceptives by six months postpartum (aRR 1.35;95% CI 1.12, 1.62). However, counselling during ANC visits was not associated with modern contraceptive uptake by either six weeks or six months postpartum. A significant proportion of women had missed the opportunity, and the postpartum modern contraceptive uptake was low. Despite these, family planning counselling prior to discharge from the facility and during child immunisation improved the postpartum modern contraceptive uptake. However, our finding revealed insufficient evidence that family planning counselling during ANC is associated with postpartum modern contraceptive uptake.

## Introduction

Postpartum contraceptive uptake reduces the risk of unintended pregnancy and associated adverse perinatal and maternal outcomes [1]. In general, women in the postpartum period–the first 12 months following delivery–wish to postpone or avoid subsequent pregnancies. However, evidence shows a significant proportion of women do not use contraceptive methods during this period partly due to the lack of access to family planning services, including counselling [2]. For example, over 60% of postpartum women in low and middle-income countries had an unmet need for contraception [3], and around 20% of women in sub-Saharan Africa (SSA) gave birth within 24 months of their previous live births [4]. Consequently, the SSA countries are the leading contributors to global maternal and perinatal morbidity and mortality [5, 6]. Fulfilling the unmet need for contraception among women in the postpartum period could avert up to 30% of maternal mortality and 10% of child mortality [1]. Family planning counselling can play a vital role in supporting women and couples to use contraceptive methods that better suit their pregnancy and health intentions [7].

The World Health Organization (WHO) recommends family planning counselling be provided during maternal health services continuum of care such as antenatal care (ANC), childbirth, and the postpartum period including postnatal care (PNC), child immunisation and well/sick baby clinics [8]. Offering integrating family planning counselling with these maternal health services provides an opportunity for the women to receive services without the need to return to the facility on a separate occasion for family planning services [9]. Increasing contraceptive uptake will also enable women to increase their participation in the workforce, which raises household income and allows them to invest in themselves and their families [9]. Although there is no standard approach to measuring family planning counselling, it can be captured through client interviews and direct observation of the provider-client interaction [10, 11]. Despite its shortcomings, interviewing clients retrospectively, whether they had received counselling during their visits to a health facility or consultation with a health care worker, is the most used approach in low-income settings for measuring counselling [12].

A study in India showed that family planning counselling during the ANC, delivery, and postpartum period improved contraceptive uptake [13]. Similarly, studies conducted in Liberia and Mali showed that women who received family planning counselling during child immunisation were more likely to use contraceptive methods than those who did not receive counselling during child immunisation [14, 15].

In order to increase the use of modern contraceptive methods, the National Guideline for Family Planning Services in Ethiopia recommends integrating family planning counselling with the various maternal and child health (MCH) services [16]. Therefore, women are expected to receive family planning counselling while visiting a health facility for ANC, particularly during the third trimester, childbirth, and in the postpartum period while visiting for PNC, sick child clinic, child immunisation, and growth monitoring [16, 17]. Despite this policy, the effect of family planning counselling on modern contraceptive uptake in Ethiopia remains inconclusive. For example, a facility-based study in Northwest Ethiopia indicated that family planning counselling during ANC improved the postpartum contraceptive uptake [18]. In contrast, a study conducted in South Ethiopia showed that counselling during the ANC visits was not associated with contraceptive uptake [19]. Therefore, this study aims to 1) determine the extent of family planning counselling provision during MCH services, 2) estimate the magnitude of postpartum modern contraceptive uptake, and 3) examine the effect of family planning counselling at different points of MCH care on postpartum modern contraceptive uptake. The study will help improve the country’s family planning program, including improving family planning counselling and postpartum contraceptive uptake, which reduces unintended pregnancy and adverse perinatal outcomes and achieves related Sustainable Development Goals.

## Methods and materials

### Study design

We used data obtained from the Performance Monitoring for Action (PMA) Ethiopia panel survey, a community-based prospective cohort study, where pregnant women enrolled at the baseline and interviewed by six weeks and six months postpartum [20, 21]. The PMA has recruited and trained resident enumerators (RE) to collect data since the beginning of the project in 2013. The baseline interview was conducted from September to December 2019. The REs regularly monitored women’s deliveries and conducted follow-up interviews approximately by six weeks and six months postpartum between March and December 2020. April-June, data collection was paused due to the COVID-19 related restrictions in the country, but data collection resumed in July.

Women’s sociodemographic characteristics and family planning counselling during ANC were collected at the baseline interview. Family planning counselling during ANC (occurring after the baseline interview) and prior to discharge, pregnancy outcomes, and contraceptive uptake prior to discharge from the facility and during the first six weeks were collected at the six-week follow-up interview. Family planning counselling during child immunisation and modern contraceptive uptake during the first six months were captured at the six-month follow-up interview. All interviews were conducted face-to-face at the respondent’s home, and all data were self-reported.

### Study setting

Ethiopia is the second-most populous African country, where more than 80% of the population lives in a rural area. It has eleven regional states and two administrative cities. This study was conducted in five regions at the time of the study, namely Tigray, Afar, Amhara, Oromia, South Nation, Nationalities and People (SNNPR), which included the two newly established regions—Sidama and Southwest Ethiopia, and one administrative city, Addis Ababa. These comprise 90% of the total Ethiopian population. A two-stage cluster sampling technique was used in the four regions, Tigray, Amhara, Oromia and SNNPR, by stratifying rural and urban residences. Two-stage cluster sampling without stratification was used in the Afar region and Addis Ababa by assuming there would not be a difference between urban and rural as the majority of the Afar population is a pastoralist community and since Addis Ababa is an urban area. First, 217 *enumeration areas* (EA) were randomly selected across the six regions. Later, 35 households were randomly selected per EA, and all women in the selected households were screened for pregnancy status at the survey time.

**Fig 1:**
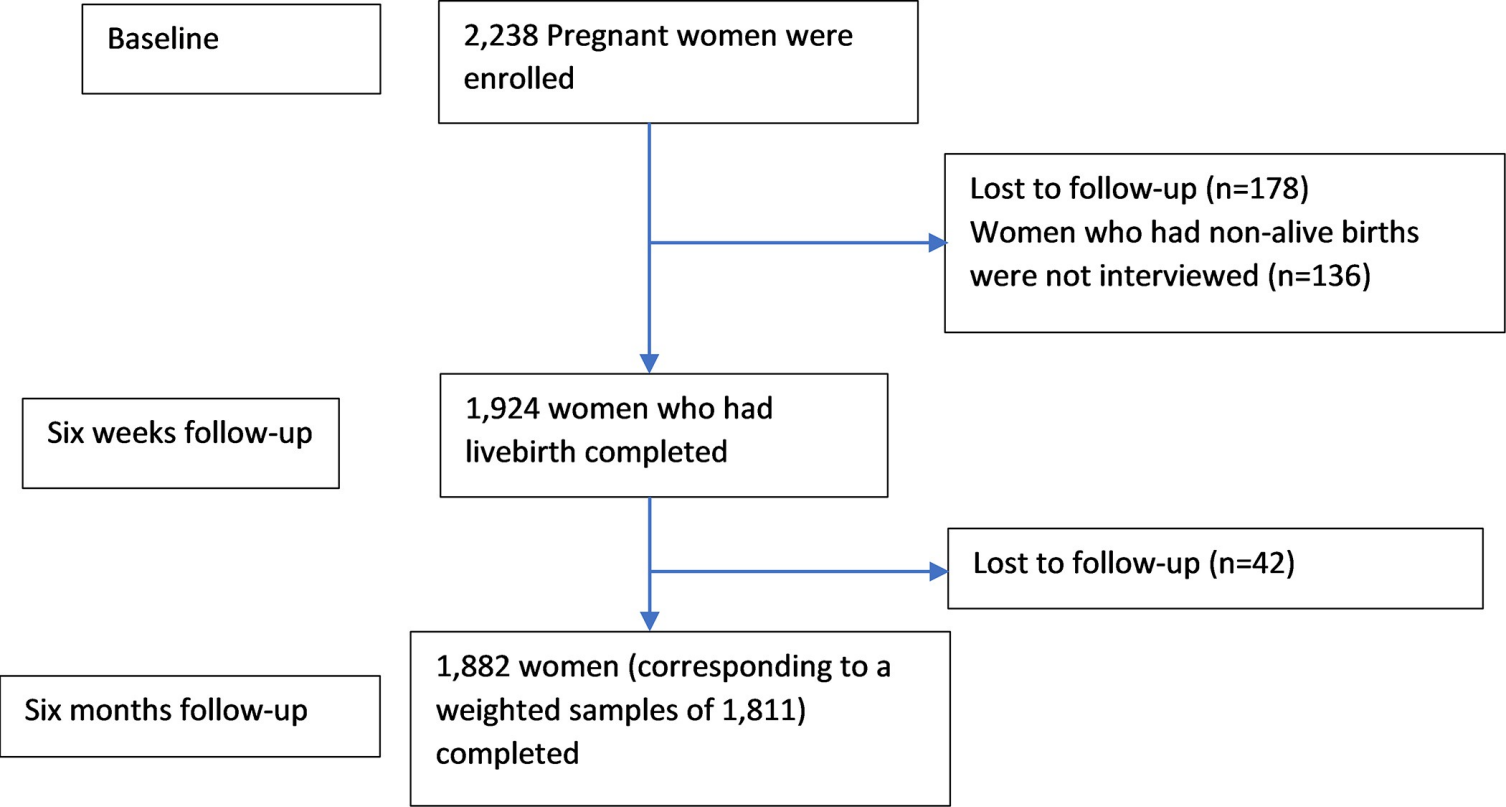
Flow diagram of participant’s’ progress through the study.

A total of 2,238 pregnant women were recruited regardless of their gestational age and interviewed during their pregnancy, at approximately six weeks and six months postpartum. Attrition was low, with 178 and 42 women being lost to follow-up at six weeks and six months, respectively. Since women who had non-live births (n = 136) were not interviewed during the follow-up interviews, 1,882 women corresponding to a weighted sample of 1,811 were included in the analysis determining the postpartum modern contraceptive uptake (**Fig 1**). Whilst women who had received MCH services, any ANC visits (n = 1,416), facility-based delivery (n = 971) and child immunisation (n = 1,486) were included for determining the receipt of family planning counselling at the corresponding service contact points. In addition, women who received the two maternal health services such as any ANC visits and facility delivery (n = 856), and women who received all those three services (n = 785) were eligible to examine the association between family planning counselling and modern contraceptive uptake at six weeks and six months postpartum, respectively (**S1 Fig**). The number of samples included in the analysis of each objective is presented in the **S1 Table**.

### Variables and measurement

#### Outcome variable

The primary outcome of this study was postpartum modern contraceptive uptake, defined as the use of any modern contraceptive methods; sterilisation (female or male), condoms (female or male), injectables, oral pills, intrauterine contraceptive device (IUCD), implants, lactational amenorrhea (LAM) and standard calendar method at the time of the survey [22]. We assessed the modern contraceptive uptake prior to discharge and during the six weeks and six months postpartum periods.

#### Exposure variables

Family planning counselling was the primary exposure variable captured from the women’s self-report. The counselling provided during the ANC was captured both at the baseline and six weeks follow-up interview, and women were asked the following a single question “during your antenatal care visit, did your provider talk with you about family planning?”. While counselling prior to discharge from the facility was captured during the six-week follow-up interview, women were asked, “before you left the facility after delivery, did a provider talk with you about using a family planning method? Moreover, information on the provision of counselling during the child immunisation was captured at the time of the six-month follow-up interview as child immunisations are provided between the first six weeks and six months postpartum. For the counselling during child immunisation, women were asked as “did you receive any family planning information during any of the immunisation visits for your baby?” The level of receipt of family planning counselling during ANC, prior to discharge, and at child immunisation were estimated from those who had received each corresponding MCH service.

#### Adjustment variables

We adjusted for women’s sociodemographic characteristics, including household wealth index, educational attainment and age; childbearing characteristics, including sex and survival of the most recent child; and health service-related factors, such as types of service providers and delivery facility, and history of complications during pregnancy and delivery. The adjustment variables are described in detail in the supplementary material (**S2 Table**).

### Statistical analysis

A weighted generalised linear model, fitted with a Poisson distribution and a log link function, was used to examine the effect of family planning counselling on postpartum modern contraceptive uptake. As the child immunisation is started after six weeks, only women who had received the two maternal health services (n = 856), ANC and health facility delivery, were included to examine the association between receipt of family planning counselling and modern contraceptive uptake by six weeks postpartum. Likewise, those who had received all the three MCH services (n = 785), including child immunisation, were included to examine the association between family planning counselling receipts and modern contraceptive uptake by six months postpartum. We adjusted for potential confounders, including women’s sociodemographic, childbearing and health service utilisation related covariates. Unadjusted and adjusted relative risk (aRR) and 95% Confidence intervals (CIs) were estimated. All statistical analyses were employed using Stata 16.1 [23].

### Ethics statement

The study sought and received ethical approval from the Johns Hopkins Bloomberg School of Public Health in Baltimore, Maryland (00009391) and Addis Ababa University, Addis Ababa, Ethiopia Institutional Review Boards (075/13/SPH). Oral informed consent was obtained from all study participants.

## Result

### Analytical cohorts

Of the 2,238 pregnant women enrolled in the baseline survey, 1,811 (81%) women completed the six-month follow-up interview. Study participants’ sociodemographic, childbearing and health service utilisation characteristics were presented in the supplementary material (**S3 Table**). Of the weighted sample (n = 1,811), 1416 women had attended at least one ANC visit during their most recent pregnancy, 971 (54%) delivered at a health facility, and 1,486 (82%) had received child immunisation. Of those who delivered at a health facility, three in five (58%) of the participants were delivered at health centres, and approximately four in ten (45%) were assisted by a Nurse/Midwife (**Table 1**).

**Table 1:** Characteristics of study participants by family planning counselling provided at the different maternal health services.

Variable	Category	Family planning counselling		
		During ANC	Prior to discharge	During child immunisation
		(N = 279)	(N = 266)	(N = 347)
		N (%)	N (%)	N (%)
Household wealth index	Lowest	117 (22)	67 (28)	119 (22)
	Middle	118 (20)	116 (30)	129 (21)
	Highest	44 (14)	83 (24)	99 (28)
Residence	Urban	54 (16)	92 (25)	99 (26)
	Rural	225 (21)	173 (29)	248 (22)
Living jurisdiction	Tigray	27 (27)	24 (31)	33 (33)
	Afar	1	-	2
	Amhara	78 (23)	44 (20)	54 (16)
	Oromia	79 (13)	107 (27)	144 (22)
	SNNPR^b^	79 (26)	66 (33)	91 (28)
	Addis Ababa	14 (26)	24 (39)	23 (36)
Women’s age in years	<25	72 (15)	95 (26)	125 (25)
	25–29	91 (21)	92 (30)	105 (23)
	30–34	60 (22)	38 (24)	64 (22)
	≥35	57 (26)	41 (29)	53 (23)
Women’s highest educational attainment	Not educated	102 (18)	71 (24)	110 (19)
	Primary	116 (21)	118 (31)	155 (27)
	Secondary	61 (20)	77 (27)	82 (26)
Religion	Orthodox	126 (22)	129 (29)	156 (26)
	Muslim	84 (17)	64 (22)	101 (20)
	Protestant	67 (22)	67 (30)	90 (25)
	Other	2	6	0
Parity	Nulliparity	40 (12)	61 (19)	85 (25)
	Primiparity	61 (21)	79 (36)	88 (28)
	Multiparity	114 (22)	85 (29)	113 (21)
	Grand multiparity	64 (24)	40 (28)	61 (22)
Pregnancy intention	Intended	179 (20)	183 (27)	220 (23)
	Unintended	100 (20)	83 (28)	127 (23)
Antenatal care provider (n = 1,416)	HP^c^	129 (16)	137 (24)	45 (26)
	HEWs^d^	49 (23)	27 (42)	174 (24)
	Both	100 (27)	68 (31)	85 (26)
Danger signs of pregnancy (n = 1,413)	No	129 (23)	118 (28)	156 (26)
	Yes	150 (18)	148 (27)	190 (22)
Type of facility where delivery was attended	Health centre	NA^e^	158 (28)	144 (28)
	Hospital		98 (26)	89 (26)
	Other facilities		10 (27)	10 (28)
Type of provider who assisted the delivery	Nurse/Midwife		127 (29)	106 (27)
	Skilled provider can’t distinguish		75 (22)	77 (25)
	Doctor		59 (34)	55 (33)
	HEW		1	1
Complication during delivery	No		144 (26)	142 (28)
	Yes		121 (29)	100 (26)
Caesarean-section delivery	No		223 (25)	217 (27)
	Yes		43 (48)	25 (30)

^a^Weighted sample

^b^South Nation, Nationalities and Peoples’ Region

^c^other trained health professionals

^d^Health extension workers

^e^Not Applicable, as delivery happened after the family planning counselling during the antenatal care.

### Family planning counselling provision

Of those women who had attended at least one ANC visit, only 279 (20%) received family planning counselling during ANC. Among those women who delivered at a health facility, 266 (27%) received family planning counselling prior to discharge from the facility and among those who received child immunisation, 347 (23%) participant women had received family planning counselling during the child immunisation. Of those who had received two maternal health services, ANC and health facility delivery, only 307 (36%) received family planning counselling; 13% at both ANC and prior to discharge, and 23% at either of the contact points. Moreover, of those who had received the three MCH services, only half of them received family planning counselling; 7% at each of the three, 15% at any two and 28% at any one contact point.

Receipt of family planning counselling during the MCH service varied by the women’s characteristics. For example, 15% of young age (<25 years) women had received family planning counselling during ANC, while 26% of older women (≥ 35 years) women had received the counselling. While one third (34%) of women whose births were attended by doctors had received family planning counselling prior to discharge, only 22% of women whose births were attended by other skilled health providers received family planning counselling (**Table 1**).

### Postpartum modern contraceptive uptake

Of the study participants, 32 (3%), 330 (18%), and 661 (36%) women were using modern contraceptive methods prior to discharge from the facility, by six weeks and six months postpartum, respectively. Implants were the most used modern contraceptive immediately after childbirth before leaving the facility, followed by oral contraceptive pills and injectables. In contrast, injectables were the most used modern contraceptive method followed by implants during the first six weeks and six months postpartum. The method mix increased from prior to discharge to six months postpartum (**S2 Fig**).

Table 2 demonstrates the study participants characteristics by modern contraceptive uptake by six weeks and six months postpartum. By six weeks postpartum, modern contraceptive uptake was lower among women in the lowest household wealth index (10%) and rural residents (14%). None of the women in the Afar region had received contraceptive methods by six weeks postpartum, whereas 50% of women in Addis Ababa used modern contraceptive methods by this time. Modern contraceptive uptake increased as women’s age and educational level increased (**Table 2**).

**Table 2:** Characteristics of study participants by modern contraceptive uptake by six weeks and six months postpartum.

Variables	Category	Modern contraceptive uptake	
		By six weeks	By six months
		N (%)	N (%)
Household wealth index	Lowest	71 (10)	160 (22)
	Middle	124 (17)	253 (35)
	Highest	134 (37)	248 (69)
Residence	Urban	135 (35)	262 (67)
	Rural	194 (14)	399 (28)
Living jurisdiction	Tigray	21 (19)	42 (37)
	Afar	0	1
	Amhara	69 (18)	152 (39)
	Oromia	120 (15)	255 (32)
	SNNPR	88 (21)	162 (38)
	Addis Ababa	32 (50)	49 (77)
Women’s age	<25 years	131 (21)	258 (42)
	25–29 years	105 (19)	222 (41)
	30–34 years	64 (18)	111 (32)
	≥35 years	30 (10)	69 (23)
Women’s highest educational attainment	Not educated	82 (11)	180 (23)
	Primary	141 (20)	284 (41)
	Secondary	107 (32)	196 (58)
Religion	Orthodox	145 (21)	307 (45)
	Muslim	77 (12)	154 (25)
	Protestant	101 (22)	190 (41)
	Other	7 (20)	10 (29)
Parity	Nulliparity	106 (26)	209 (51)
	Primiparity	93 (26)	180 (50)
	Multiparity	103 (16)	206 (31)
	Grand multiparity	28 (7)	65 (17)
Pregnancy intention	Intended	232 (20)	446 (40)
	Unintended	98 (15)	215 (33)
Antenatal care provider	HEWs^1^	36 (17)	65 (31)
	HP^2^	182 (22)	348 (42)
	Both	70 (19)	155 (42)
Danger signs of pregnancy	No	160 (22)	305 (41)
	Yes	170 (16)	356 (33)
Type of facility where they gave delivery	Hospital	124 (34)	219 (59)
	Health centre	115 (21)	246 (44)
	Other facilities	12 (33)	25 (67)
Type provider assisted the delivery	Nurse/Midwife	114 (26)	218 (50)
	Skilled provider can’t distinguish	77 (22)	167 (48)
	Doctor	61 (35)	107 (62)
	HEW	1	-
Complication during delivery	No	140 (25)	291 (53)
	Yes	112 (27)	199 (48)
Caesarean-section delivery	No	218 (25)	430 (49)
	Yes	34 (38)	60 (67)

^1^ HEWs-health extension workers

^2^ HP-other trained health professionals

### Association between family planning counselling and postpartum modern contraceptive uptake

The result of the unadjusted model indicated that receipt of family planning counselling during the relevant MCH services were associated with increasing modern contraceptive uptakes by six weeks and six months. Similarly, the adjusted model showed that family planning counselling during child immunisation was associated with increased modern contraceptive uptake by six months (aRR 1.35; 95% CI 1.12, 1.62). Although the result of the adjusted model demonstrated that receipt of family planning counselling prior to discharge was associated with increased modern contraceptive uptake by six weeks (aRR 1.25: 95% CI 0.94, 1.65) and six months (aRR 1.07; 95% CI 0.90, 1.27), its effect estimate was not precise (wide CIs). Nevertheless, the result of the adjusted model revealed that receipt of family planning counselling during ANC was not associated with modern contraceptive uptake both by six weeks (aRR 0.97; 95% CI 0.71, 1.32) and six months (aRR 0.96; 95% CI 0.82, 1.12) [**Tables 3 and 4**].

**Table 3:** Association between family planning counselling during the maternal health services and modern contraceptive uptake at six weeks postpartum.

Timing of family planning counselling	Response	Modern contraceptive uptake at six weeks postpartum		RR (95% CI)	
		No	Yes	Unadjusted model (n = 855)	Adjusted model^a^ (n = 849)
Antenatal care	No	499 (74)	171 (26)	1	1
	Yes	135 (73)	49 (27)	1.04 (0.74, 1.48)	0.97 (0.71, 1.32)
Prior to discharge	No	477 (76)	146 (24)	1	1
	Yes	157 (68)	74 (32)	**1.36 (1.03, 1.81)**	1.25 (0.94, 1.65)

^a^ Model adjusted for household wealth index, residence, living jurisdiction, age, educational attainment, religion, parity, pregnancy intention, history of pregnancy complication, type of antenatal care providers, type of facility where given delivery, type of provider assisted the delivery, history of any complication during delivery, caesarean-section delivery, child survival, child sex, and a plurality.

**Table 4:** Association between family planning counselling during the maternal health services and modern contraceptive uptake at six months postpartum.

Timing of family planning counselling	Response	Modern contraceptive uptake at six months postpartum		RR (95% CI)	
		No	Yes	Unadjusted model (n = 785)	Adjusted model^a^ (n = 778)
Antenatal care	No	289 (47)	326 (53)	1	1
	Yes	82 (48)	88 (52)	0.97 (0.81, 1.18)	0.96 (0.82, 1.12)
Prior to discharge	No	278 (49)	289 (51)	1	1
	Yes	93 (43)	125 (57)	1.12 (0.95, 1.32)	1.07 (0.90, 1.27)
Child immunisation	No	296 (52)	270 (48)	1	1
	Yes	75 (34)	144 (66)	**1.38 (1.14, 1.66)**	**1.35 (1.12, 1.62)**

^a^ Model adjusted for household wealth index, residence, living jurisdiction, age, educational attainment, religion, parity, pregnancy intention, history of pregnancy complication, type of antenatal care providers, type of facility where given delivery, type of provider assisted the delivery, history of any complication during delivery, caesarean-section delivery, child survival, child sex, plurality, resumption of menstrual cycle and the length of a wait before resuming sexual activity after the recent birth.

## Discussion

This study examined the association between family planning counselling and postpartum modern contraceptive uptake using a nationally representative community based prospective cohort study. It is also assessed the proportion of women receiving family planning counselling during the MCH services and the prevalence of postpartum modern contraceptive uptake.

Consistent with the national and international recommendations, this study revealed that receiving family planning counselling integrated with MCH services after delivery improved the modern contraceptive uptake during the postpartum period. Women who received family planning counselling prior to discharge were 25% and 7% more likely to use modern contraceptive methods in the first six weeks and six months of postpartum, respectively. This is consistent with studies conducted in Ethiopia [19] and Rwanda [24]. As it is not exactly known when women’s menses return after childbirth, it is recommended that family planning counselling and services be provided to women giving birth at health facilities prior to discharge to increase women’s awareness of the benefits of spacing pregnancy and improve women’s knowledge and attitude towards modern contraceptive methods [25]. Our study also showed that women who received family planning counselling during child immunisation were 35% more likely to use modern contraceptives within the first six months postpartum. This finding is consistent with studies conducted in Malawi and Liberia that indicated family planning counselling during child immunisation was associated with increased modern contraceptive uptake [14, 15]. This might be because women have better knowledge as they could have been counselled at multiple contacts, including ANC, childbirth, and immunisation. In Ethiopia, routine child immunisation is provided at health facilities as well as at outreach and mobile sites that are relatively nearby and convenient for the community at six weeks, ten weeks, 14 weeks and nine months [26], which could allow women to receive family planning counselling at multiple contacts, and thereby they became motivated to use contraceptive methods. In addition, as women would have greater access to child immunisation clinics than family planning services clinics [27], counselling during immunisation could help reach large numbers of postpartum women.

On the other hand, it could also be because most women have resumed their sexual activity and menses and feel they are at risk of unintended pregnancy [28, 29]. Our findings may support previous studies indicating that the return of sexual activity and menstrual cycle following childbirth were the strongest predictors for adopting modern contraceptives in Ethiopia [19, 30, 31]. These findings underscore the importance of improving the integration of family planning counselling into relevant MCH services.

However, our finding revealed insufficient evidence that family planning counselling during ANC visits was associated with modern contraceptive uptake by six weeks and six months postpartum. This is consistent with studies conducted in South Ethiopia [19] and Kenya [32]. However, other studies evaluating the effect of family planning counselling during ANC on postpartum modern contraceptive uptake have reported a positive association [18, 30, 31, 33]. For example, a study conducted in Northern West Ethiopia indicated that family planning counselling during ANC was associated with increasing modern contraceptive uptake by six weeks postpartum [18]. Similarly, a study in Nepal indicated that family planning counselling during ANC has improved postpartum contraceptive uptake [34]. The lack of consistency between studies investigating an association between family planning counselling during ANC and postpartum modern contraceptive uptake may be related to differences in the quality of the family planning counselling provided.

As per the Ethiopian Ministry of Health recommendations, pregnant women are expected to receive a variety of health advice during ANC, including family planning counselling [16, 17, 35]. Nevertheless, if pressed for time, health care workers may not offer family planning counselling during ANC, assuming counselling could be provided after delivery, and instead concentrate on providing information more salient to the pregnancy and delivery. Consequently, women might receive inadequate family planning counselling during ANC. Even if counselling is provided during ANC, the ability to recall what has happened during pregnancy could be another factor. As pregnant women are mainly concerned about their health status and birth outcome, they might not pay attention to family planning counselling and recall the information that has been given during pregnancy. As the effect of family planning counselling during ANC on postpartum modern contraceptive uptake remains inconclusive, we suggest further studies to qualitatively investigate women’s and health care workers’ perceptions on this issue.

The present study demonstrated that family planning counselling provision during the ANC, prior to discharge and child immunisation is low, 20%, 27% and 23%, respectively. The family planning during ANC is lower than previous studies conducted in Ethiopia [18, 19, 36, 37]. Earlier studies conducted at district levels in Ethiopia indicated that family planning counselling coverage during ANC ranges from 24–74% [18, 36, 37]. However, the coverage of family planning counselling prior to discharge was higher than in previous studies [36, 38]. The discrepancies could be due to sociodemographic differences in the study participants. Most of the earlier studies were conducted at a specific district level with a facility-based and urban setting [18, 36, 37], while the present study was conducted at six regions, which comprises more than 90% of the total population, and a community setting, including urban and rural resident women. As no previous study has investigated the delivery of family planning counselling at child immunisation visits, we have no comparator for this outcome. In general, however, the family planning counselling provided at different contact points in Ethiopia is low. Although it is not well investigated why health care workers in Ethiopia do not provide integrated family planning counselling services, low awareness among health care workers of the National Guideline for Family Planning Services, lack of motivation, patient overload, and other infrastructural barriers might contribute to these high rates of missed opportunity [39]. Along with this, lack of transportation due to the travel restrictions implemented in the country as a consequence of the COVID-19 pandemic and fear of acquiring the infection might have contributed to the low family planning counselling coverage reported in this study [30]. Studies in Ethiopia indicated that women faced challenges in accessing family planning services because of COVID-19 [30, 40].

Our findings also indicate that the modern contraceptive uptake during the postpartum period was low; 3% prior to discharge, 18% by six weeks, and 36% by six months. This was similar to earlier studies conducted in Ethiopia; Bahri Dar (19% by six weeks) and South Ethiopia (45% by six months) [18, 19]. The high rate of missed opportunities for family planning counselling is likely to have contributed to the low modern contraceptive uptake reported in our study. Therefore, many women are exposed to the risk of unintended and closely spaced pregnancy and the associated increased risk of adverse pregnancy consequences. The sociocultural norms and low-risk perception for unintended pregnancy could also have contributed to the low modern contraceptive uptake during the postpartum period [41–44]. Ethiopian women typically have low autonomy in the household, including not deciding about their fertility matters [41]. Additionally, the role of religion in hindering modern contraceptive uptake is paramount [42, 43]. Prior studies have reported that many women in Ethiopia do not consider themselves to be at risk of unintended pregnancy until they resume their menstrual cycle following delivery [44].

It can be assumed that a significant number of women in Ethiopia have missed the opportunity to receive family planning counselling despite the fact that women are receiving MCH health services at several contact points and that it is Government policy to integrate family planning counselling into these services. Our findings indicate that the Ethiopian Government should make a strong effort to improve the coverage of family planning counselling and postpartum modern contraceptive uptake through full implementation of their Family Planning Guideline and further strengthening and promoting family planning counselling integration. Additionally, the health care workers also need to adhere to the National guideline and procedures. Further research should be undertaken to investigate why health care workers in Ethiopia are not providing integrated family planning counselling to all women.

The main strength of this study is the cohort design in which the pregnant women were followed up to six months postpartum period, allowing the collection of the exposure variable prior to the outcome and allowing the trend of the modern contraceptive uptake along the postpartum period to be investigated. However, our study is not free of limitations. Firstly, the provision of family planning counselling was ascertained only for the most recent pregnancy. Nevertheless, women might have received counselling during previous pregnancies or from other sources such as mass media or social media, which could improve women’s knowledge of the use of postpartum modern contraceptives. In another way, even during the current pregnancy, women could have received family planning counselling more than once at specific maternal and child health services. However, in the PMA survey, women were not asked about the frequency of counselling, which could limit our analysis to investigate further if the frequency of counselling has improved postpartum modern contraceptive uptake. Secondly, as our counselling was measured from a single self-reported question, it is not easy to describe the depth and breadth of the counselling and thus could be subject to recall bias. Nevertheless, as there is no validated instrument to measure family planning counselling so far, asking a single question has been used for measuring counselling. Thirdly, as the study was conducted among women who had received the MCH services, our findings may not be generalisable to all women because women who had visited a health facility for the MCH services could be different from those who did not receive the services. Therefore, the proportion of women receiving family planning services and the prevalence of postpartum modern contraceptive uptake may, in fact, be lower than what is reported here. Finally, we did not assess the supply-related factors, including the availability of contraceptive methods, knowledge and skills of health care workers in counselling, the quality of family planning counselling, and client satisfaction with counselling services.

## Conclusion

Although family planning counselling should be fully integrated during MCH services, the study revealed a high rate of missed opportunities for family planning counselling during maternal and child health services and a low prevalence of postpartum modern contraceptive uptake in Ethiopia. Despite these, family planning counselling prior to discharge from the facility and child immunisation was associated with increased modern contraceptive uptake along the postpartum periods. In contrast, we found insufficient evidence that family planning counselling during ANC visits was associated with postpartum modern contraceptive uptake.

Our finding highlights the need for further strengthening and promotion of the integration of family planning counselling with maternal and child health services, more importantly with child immunisation. This could significantly help increase the coverage of family planning counselling and contraceptive uptake, reducing the incidence of unintended pregnancy, thereby contributing to saving the lives of mothers and improving birth outcomes.

## Supporting information

S1 Fig Flow diagram shows the number of women who received different maternal and child health services. (TIF)

S2 Fig The types of modern contraceptive methods used across the postpartum period. IUCD-Intrauterine Contraceptive Device, EC-Emergency Contraceptives, LAM-Lactational Amenorrhea Method, OCP-Oral Contraceptive Pills, Std-Standard.
(TIF)

S1 Table Lists of adjustment variables and their definition. (DOCX)

S2 Table Objectives of the study and number of included samples. (DOCX)

S3 Table Sociodemographic, childbearing and health service utilisation characteristics of study participants. (DOCX)

S1 Appendix STROBE checklist. (DOCX)

S2 Appendix Questionnaires. (DOCX)
