# Supplementary figures and images for: The role of family planning counselling during maternal and child health services in postpartum modern contraceptive uptake in Ethiopia: A national longitudinal study
Source: PLOS Glob Public Health. 2022 Aug 3;2(8):e0000563. doi: 10.1371/journal.pgph.0000563 (PMC10021256; doi:10.1371/journal.pgph.0000563)

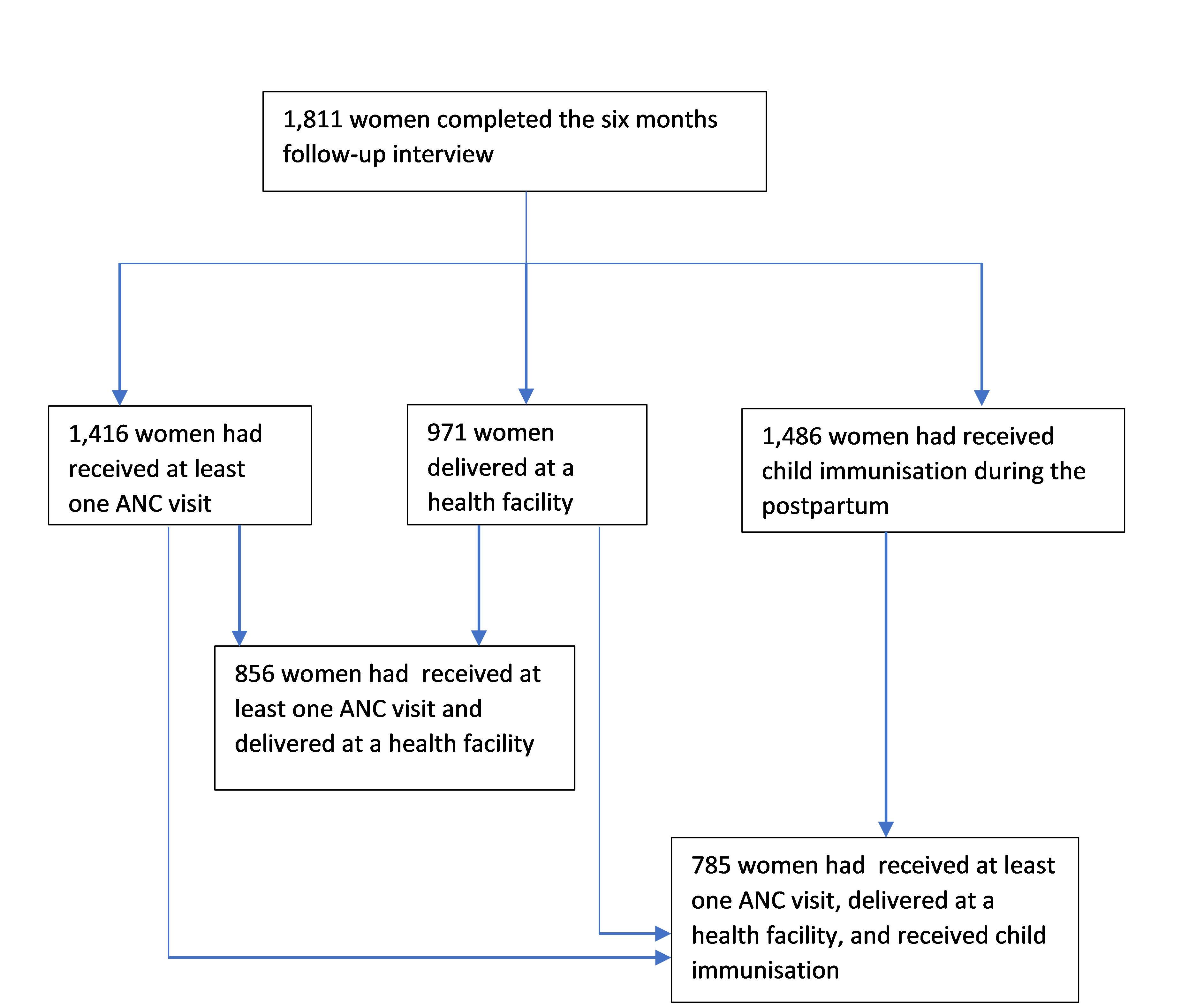

Supplement: S1 Fig — (TIF) [file pgph.0000563.s001.tif]

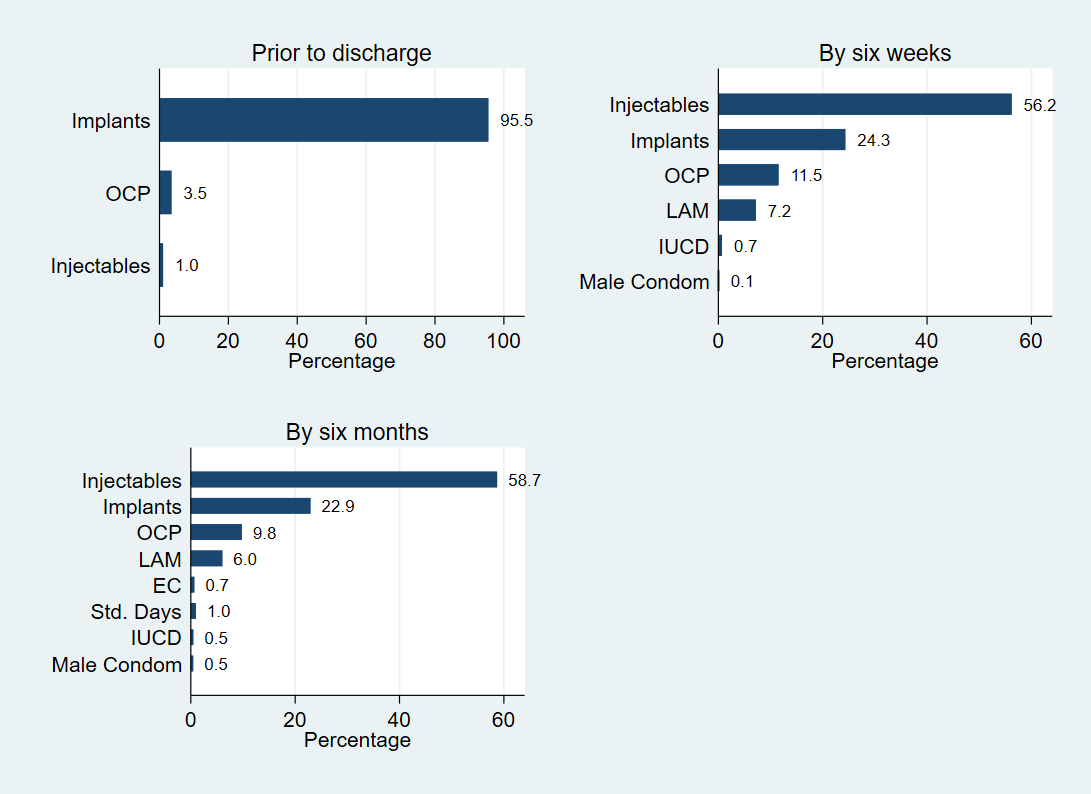

Supplement: S2 Fig — IUCD-Intrauterine Contraceptive Device, EC-Emergency Contraceptives, LAM-Lactational Amenorrhea Method, OCP-Oral Contraceptive Pills, Std-Standard. (TIF) [file pgph.0000563.s002.tif]
