# Supplementary material for: The role of family planning counselling during maternal and child health services in postpartum modern contraceptive uptake in Ethiopia: A national longitudinal study
Source: PLOS Glob Public Health. 2022 Aug 3;2(8):e0000563. doi: 10.1371/journal.pgph.0000563 (PMC10021256; doi:10.1371/journal.pgph.0000563)
Supplement: S1 Table — (DOCX) [file pgph.0000563.s003.docx]

**S1 Table . Lists of adjustment variables and their definition**

| **Covariate** | **Definition** |
| --- | --- |
| Household wealth index | The household wealth index was calculated from the household's ownership assets, materials used to construct the house, types of water access and sanitation facilities using the principal component analysis (PCA), considering the urban-rural differences. Later, the wealth index score was divided into three equal parts labelled as lowest, middle and highest |
| Residence (urban vs rural) | Participant place of residence coded as urban or rural. Urban was defined as a locality with 2,000 or more inhabitants. Moreover, all administrative capitals, including Region, Zone and Woreda, and localities in which urban dwellers’ associations were established were considered urban residences, irrespective of the population size. Rural residences comprise all areas not classified as urban. |
| Living of jurisdiction | This study was conducted in five regional states and one city administration, including Tigray, Afar, Amhara, Oromia, SNNPR and Addis Ababa |
| Maternal age at enrolment | Maternal age at birth of the preceding child was categorised into <25 years, 25-29 years, 30-34 years and ≥35 years |
| Maternal education | Maternal educational status was categorised into not educated, primary, secondary, and higher (technical and vocational, and above) |
| Religion | Women's religion was classified into Orthodox, Muslim, Protestant, and others, including Catholic, Wakefetah, Traditional followers |
| Marital status | Women's marital status was categorised as married vs not married, including single, widowed, and divorced |
| Parity | As parity was captured during the baseline interview, it was considered excluding the recent birth and categorised as nulliparity (parity 0), primiparity (parity 1), multiparity (parity 2-4) and grand multiparity (parity ≥5) |
| Pregnancy intention | The pregnancy intention of the recent pregnancy was captured as 'then', 'later', and 'not at all'. We recategorised 'then' as intended pregnancy and 'later' and 'not at all' as unintended pregnancy. |
| Intimate partner violence (IPV) during pregnancy | Conflict and Tactics Scale (CTS-2) consisting of ten item questions, was used to assess any intimate partner violence during the recent pregnancy. Women who reported experiencing any, either physical and /or sexual, violence from their intimate partner during the recent pregnancy were labelled as having IPV and otherwise as not having IPV. |
| Antenatal Care (ANC) visits | Women were asked whether they received ANC from a health extension worker (HEW), either at a health post or at home, or care from a professional health care provider other than a HEW. Women who responded "no" to receiving any ANC from either a HEW or another health care professional were considered as having no ANC visits. Women who had received any ANC from either a HEW or another health care professional were considered as having ANC visits |
| Types of ANC providers | The providers who assisted women’s ANC visits were categorised as health care providers, health extension workers (HEWs) or both from health care providers and HEWs |
| Complications during the recent pregnancy | Women reporting any of the danger signs of pregnancy; *Severe headache with blurred vision, high blood pressure, oedema face/feet/body, convulsion/fits, vaginal bleeding before delivery, high fever, abnormal vaginal discharge (foul-smelling/dark), lower abdominal pain, worsening vision, particularly at night*, were considered to have experienced pregnancy complications |
| Type of health facilities where given birth | The types of health facilities where women delivered were categorised as government health centres, government hospitals, and others, including private and non-governmental health facilities |
| Birth assistant | The types of health care providers who assisted the recent delivery were grouped into Nurse/midwife, skilled attendant can’t distinguish, Doctors and HEWs. |
| Complications during delivery | Women reporting any of the following during delivery; *severe bleeding, Leaking/rupture of membrane and no labour pain for >24 hours, Leaking/rupture of membrane before nine months, malpresentation, prolonged labour (<12 hours), and convulsion/fits,* were considered to have experienced a complication during delivery. |
| Caesarean-section delivery for the recent birth | Women were asked whether the recent birth was delivered by caesarean section and recorded as yes vs no |
