# Supplementary material for: The role of family planning counselling during maternal and child health services in postpartum modern contraceptive uptake in Ethiopia: A national longitudinal study
Source: PLOS Glob Public Health. 2022 Aug 3;2(8):e0000563. doi: 10.1371/journal.pgph.0000563 (PMC10021256; doi:10.1371/journal.pgph.0000563)
Supplement: S2 Table — (DOCX) [file pgph.0000563.s004.docx]

S2 Table: Objectives of the study and number of included samples

| **Objectives** | **Samples** |
| --- | --- |
| 1. Determine the level of family planning counselling during | Women who had received the corresponding MCH services |
| 1. ANC | Women had received at least one ANC visit (n=1416) |
| 1. Prior to discharge from the facility | Women delivered at health facility (n=971) |
| 1. Child immunisation | Women had received child immunisation during the postpartum (n=1486) |
| 1. Determine the postpartum modern contraceptive uptake | All postpartum women (n=1811) |
| 1. Examine the association between family planning counselling and postpartum modern contraceptive uptakes |  |
| 1. By six weeks postpartum | Women had received at least one ANC visit and delivered at health facility (n=856) |
| 1. By six months postpartum | Women had received at least one ANC visit, delivered at a health facility, and received child immunisation (n=785) |
