# Supplementary material for: The role of family planning counselling during maternal and child health services in postpartum modern contraceptive uptake in Ethiopia: A national longitudinal study
Source: PLOS Glob Public Health. 2022 Aug 3;2(8):e0000563. doi: 10.1371/journal.pgph.0000563 (PMC10021256; doi:10.1371/journal.pgph.0000563)
Supplement: S3 Table — (DOCX) [file pgph.0000563.s005.docx]

S3 Table. Sociodemographic, childbearing and health service utilisation characteristics of study participants

| **Variable** | **Category** | **Total**  **N=1,811^a^** |
| --- | --- | --- |
|  |  |  |
| Household wealth index | Lowest | 733 (40) |
|  | Middle | 719 (40) |
|  | Highest | 359 (20) |
| Residence | Urban | 389 (22) |
|  | Rural | 1,421 (78) |
| Living jurisdiction | Tigray | 111 (6) |
|  | Afar | 35 (2) |
|  | Amhara | 388 (21) |
|  | Oromia | 794 (44) |
|  | SNNPR^b^ | 419 (23) |
|  | Addis Ababa | 64 (4) |
| Women’s age in years | <25 | 614 (34) |
|  | 25-29 | 544 (30) |
|  | 30-34 | 348 (19) |
|  | ≥35 | 304 (17) |
| Women’s highest educational attainment | Not educated | 773 (43) |
|  | Primary | 698 (39) |
|  | Secondary | 340 (19) |
| Religion | Orthodox | 689 (38) |
|  | Muslim | 623 (34) |
|  | Protestant | 465 (26) |
|  | Other | 34 (2) |
| Parity | Nulliparity | 408 (23) |
|  | Primiparity | 360 (20) |
|  | Multiparity | 659 (36) |
|  | Grand multiparity | 383 (21) |
| Pregnancy intention | Intended | 1,146 (63) |
|  | Unintended | 662 (37) |
| Antenatal care provider (n=1,416) | HP^c^ | 834 (59) |
|  | HEWs^d^ | 214 (15) |
|  | Both | 368 (26) |
| Danger signs of pregnancy (n=1,413) | No | 568 (40) |
|  | Yes | 845 (60) |
| Type of facility where delivery was attended | Health centre | 562 (59) |
|  | Hospital | 372 (38) |
|  | Other facilities | 38 (4) |
| Type of provider who assisted the delivery | Nurse/Midwife | 439 (46) |
|  | Skilled provider can’t distinguish | 350 (36) |
|  | Doctor | 173 (18) |
|  | HEW | 4 |
| Complication during delivery | No | 553 (57) |
|  | Yes | 418 (43) |
| Caesarean-section delivery | No | 882 (91) |
